# Integrated Text Mining and Chemoinformatics Analysis Associates Diet to Health Benefit at Molecular Level

**DOI:** 10.1371/journal.pcbi.1003432

**Published:** 2014-01-16

**Authors:** Kasper Jensen, Gianni Panagiotou, Irene Kouskoumvekaki

**Affiliations:** 1 Center for Biological Sequence Analysis, Department of Systems Biology, Technical University of Denmark, Kemitorvet, Lyngby, Denmark; 2 School of Biological Sciences, The University of Hong Kong, Hong Kong; Stanford University, United States of America

## Abstract

Awareness that disease susceptibility is not only dependent on genetic make up, but can be affected by lifestyle decisions, has brought more attention to the role of diet. However, food is often treated as a black box, or the focus is limited to few, well-studied compounds, such as polyphenols, lipids and nutrients. In this work, we applied text mining and Naïve Bayes classification to assemble the knowledge space of food-phytochemical and food-disease associations, where we distinguish between disease prevention/amelioration and disease progression. We subsequently searched for frequently occurring phytochemical-disease pairs and we identified 20,654 phytochemicals from 16,102 plants associated to 1,592 human disease phenotypes. We selected colon cancer as a case study and analyzed our results in three directions; i) one stop legacy knowledge-shop for the effect of food on disease, ii) discovery of novel bioactive compounds with drug-like properties, and iii) discovery of novel health benefits from foods. This works represents a systematized approach to the association of food with health effect, and provides the phytochemical layer of information for nutritional systems biology research.

## Author Summary

Until recently diet was considered a supplier of energy and building blocks for growth and development. However, current research in the field suggests that the complex mixture of natural compounds present in our food has a variety of biological activities and plays an important role for health maintenance and disease prevention. The mixture of bioactive components of our diet interacts with the human body through complex processes that modify network function and stability. In order to increase our limited understanding on how components of food affect human health, we borrow methods that are well established in medical and pharmacological research. By using text mining in PubMed abstracts we collected more than 20,000 diverse chemical structures present in our diet, while by applying chemoinformatics methods we could systematically explore their numerous targets. Integrating the above datasets with food-disease associations allowed us to use a statistical framework for identifying specific phytochemicals as perturbators of drug targets and disease related pathways.

## Introduction

The increasing awareness of health and lifestyle in the last decade has brought significant attention from the public media to the role of diet. Typically, specific diets or single foods are associated with health and disease states through *in vivo* studies on humans or animal models, where the response of selected phenotypes, e.g. up-regulation or down- regulation of certain genes, is being monitored [1], [2]. Observational studies on populations with specific food preferences may also provide statistical evidence for the absence or prevalence of certain diseases in connection to certain dietary habits [3]. Even though these approaches have offered some useful insights for specific food types, they are frequently inconclusive due to small cohorts or limited focus both on the diet and the disease space. Most importantly, observations remain on the phenotypic layer, since diet is treated as a black box, when it comes to its molecular content. In the emerging field of systems chemical biology [4] research is moving towards the network-based study of environmental exposures, (e.g. medicine, diet, environmental chemicals) and their effect on human health [5]. We believe that this shift in paradigm, where one considers the system of the molecular components of diet and their interplay with the human body, will build the basis for understanding the benefits and impact of diet on our health that will enable the rational design of strategies to manipulate cell functions through what we eat [6], [7]. However, to interpret the biological responses to diet, as well as contribute to the evidence in assigning causality to a diet-disease association, we need first to overcome the major barrier of defining the small molecule space of our diet. By assembling all available information on the complex chemical background of our diet, we can systematically study the dietary factors that have the greatest influence, reveal their synergistic interactions, and uncover their mechanisms of action.

In the present work we carried out text mining to collect in a systematic and high-throughput way all available information that links plant-based diet (fruits, vegetables, and plant-based beverages such as tea, coffee, cocoa and wine) with phytochemical content, i.e. primary and secondary metabolites, and human disease phenotypes. There are two reasons for focusing on the plant-based diet: *(1)* there is well established knowledge on the importance of fruit- and vegetable-rich diet in relation to human health e.g. nutraceuticals, antibiotics, anti-inflammatory, anti-cancer, just to name a few [8]–[13]; *(2)* the huge diversity of the phytochemical space offers a fertile ground for integrating chemoinformatics with statistical analysis to go beyond the existing knowledge in the literature and suggest new associations between food and diseases.

Our text-mining strategy, based on dictionaries from the argument browser Reflect [14], Natural Language Processing (NLP) and Naive Bayes text classification [15], [16], goes beyond mere retrieval of diet - disease associations, as it further assigns a positive or negative impact of the diet on the disease. With this work we aim to demonstrate how data from nutritional studies can be integrated in systems biology to boost our understanding of how plant-based diet supports health and disease prevention or amelioration. This wealth of knowledge combined with chemical and biological information related to food could pave the way for the discovery of the underlying molecular level mechanisms of the effect of diet on human health that could be translated into public health recommendations.

## Results

### Mining the phytochemical space

We extracted by text mining plant - phytochemical associations from 21 million abstracts in PubMed/MEDLINE, covering the period 1908–2012. We used relation keyword co-occurrences between plant names (both common names and scientific names) and small compound names and synonyms. First, the chemical name entities and plant name entities were recognized using a set of simple recognition rules. Then, a training set was manually compiled with abstracts mentioning plant - phytochemical pairs. Finally, a Naïve Bayes classifier was trained to correctly recognize and extract pairs of phytochemicals and plants that contain them. The performance of the classifier was quantitatively estimated to 88.4% accuracy and 87.5% F1-measure on an external test set of 250 abstracts.

When the classifier was applied to the raw text of PubMed/MEDLINE, it associated 23,137 compounds to 15,722 plants – of which, approximately 2,768 are edible – through 369,549 edges. Since the total number of natural compounds discovered so far from all living species is estimated to be approximately 50,000 [17], the retrieval of 23,137 phytochemicals solely by extraction of information from raw text of titles and abstracts in the PubMed domain provides a unique platform for obtaining a holistic view of the effects of our diet on health homeostasis.

In order to collect all relevant available information for subsequent analyses, we integrated the data we collected via text mining with the Chinese Natural Product Database [18] (CNPD) and an Ayurveda [19] data set that we have previously curated in house. CNPD, which is a commercial, manually curated database, contains information on 16,876 unique compounds from 5,182 plant species associated through 21,172 edges. The Ayurveda data set includes information on 1,324 phytochemicals and 189 plants. After merging these two sources with the text-mined data and removing redundant information, we ended up with 36,932 phytochemicals and 16,102 plants. What further adds value to this pool of data is that all 36,932 compounds are encoded in Canonical SMILES and linked to a unique chemical structure, which allows the application of chemoinformatics tools for interrogating the human protein and disease space that these compounds may have an effect on.

**Figure 1:**
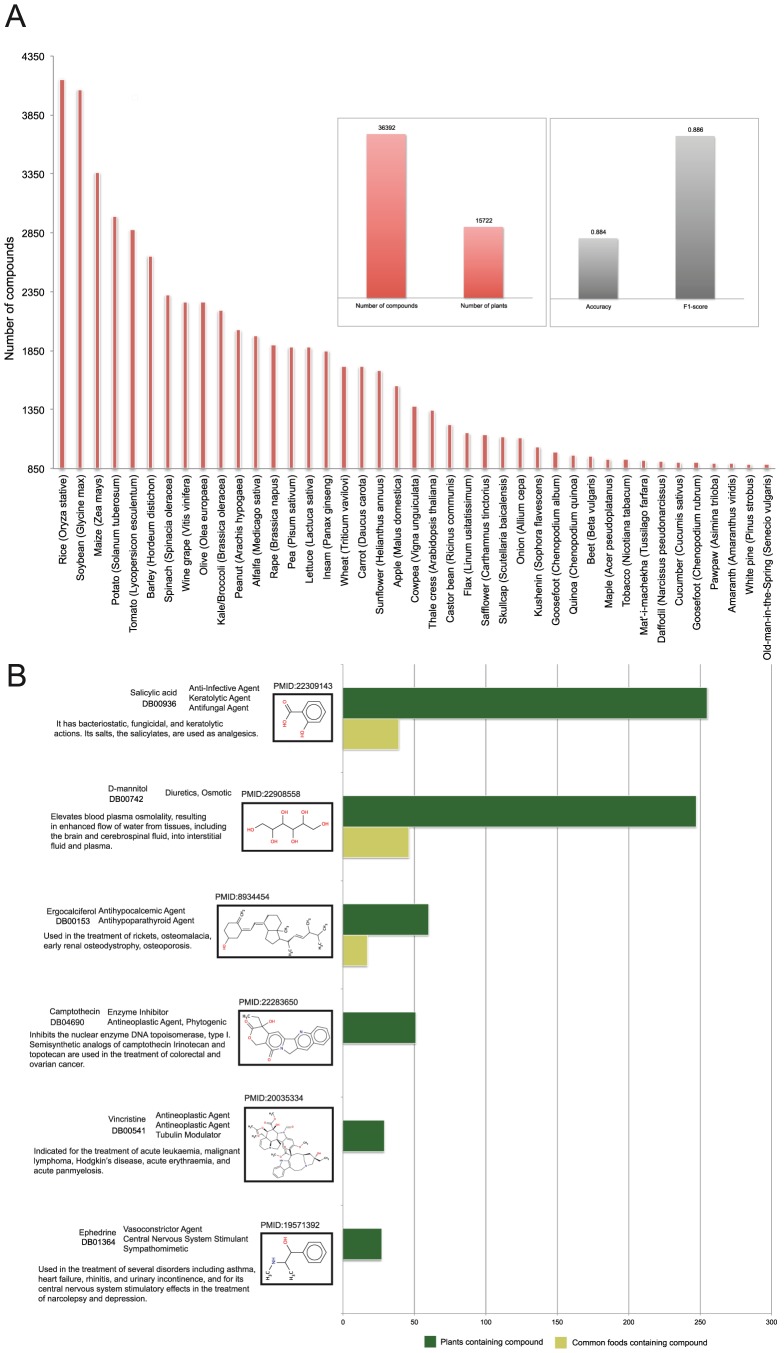
Distribution of plant species and recorded phytochemicals. a) Distribution of phytochemicals on the plant space. Rice, soybean, maize and potato are the plants with the most recorded phytochemicals: 4,155, 4,064, 3,361 and 2,988 compounds respectively. b) Structures of representative phytochemicals that have made the way to the pharmacy shelves and their occurrence in respective edible sources.

Figure 1A shows the most well studied edible plants and the number of phytochemicals identified in each of them. Rice has the highest number of recorded phytochemicals (4,155 compounds), followed by soybean (4,064 compounds), maize (3,361 compounds) and potato (2,988 compounds). Figure 1B shows representative phytochemicals from our retrieved data that have made it all the way to the pharmacy shelves or have served as lead structures for drug development. Camptothecin is a natural compound that has lead to the semisynthesis of the analogues irinotecan and topotecan, two antineoplastic enzyme inhibitors that are currently used in the treatment of colorectal and ovarian cancer, respectively. As camptothecin is highly cytotoxic, we have not encountered any common foods within the list of plants that contain it. Ergocalciferol (vitamin D2), on the other hand, has been traced in numerous plant sources, many of which are common foods, such as tomato, cacao and alfalfa. Ergocalciferol is an approved nutraceutical compound found in the market under various brand names that is used in the treatment of diseases related to vitamin D deficiency, such as hypocalcemia, rickets and osteomalacia.

Figure 1B brings also to light that natural compounds are comonly encountered in more than one plant, or family of plants. Previous studies have indicated that there are no consistent trends as to whether phytochemicals can be used as taxonomic markers or may occur in several unrelated plant families [8], [20]. With this question in mind, we decided to examine how the 36,932 phytochemicals are distributed among neighboring and ancestral taxa and whether there are clusters of certain phytochemicals at specific parts of the taxonomy. Overrepresentation of phytochemicals on the taxonomy was calculated by using Fishers exact test, following the Benjamini-Hochberg procedure with a 5% False Discovery Rate [21]. Our analysis showed that only 8% of all phytochemicals are localized on certain parts of the taxonomy (Figure S1 and Table S1). For example the family of *Fabales – Fabaceae – Lens*, which includes lentils, and the *Sapindales – Rutaceae – Citrus* linkage, which includes orange, contain 60 out of 562 compounds and 42 out of 214 compounds, respectively (*p*-value<10^−4^) that are not found anywhere else on the taxonomy. On the other hand, compounds such as β-sitosterol, palmitic acid and catechin are spread all over the taxonomy (*p*-value<10^−4^). A possible interpretation of this finding is that the synthesis of small compounds in plants is mainly defined by short-term regulatory than long-term evolutionary adaptation to the environment.

### Association of food with disease prevention or progression

To systematically associate plant-based diet with health effect we extracted by text mining plant - disease associations from 21 million abstracts in PubMed/MEDLINE, covering the period 1908–2012. In this manner we associated 7,106 plant species, 2,768 of which edible, with 1,613 human disease phenotypes. The performance of the classifier was quantitatively estimated to 84.5% accuracy and 84.4% F1-measure on an external test set of 250 abstracts. Natural Language Processing allowed us to add directionality to these associations, an extremely valuable feature for dietary recommendations. This enabled us not only to link a certain food to a disease, but also to characterize the association as being positive (food associated with disease prevention or amelioration) or negative (food associated with disease progress). Together with the temporal parameter that is included in the text-mined data (date of publication of articles that associate food to disease), one can make interesting observations as to when scientists began showing interest in the health effect of food and how opinion regarding a certain food has been varying throughout time.

**Figure 2:**
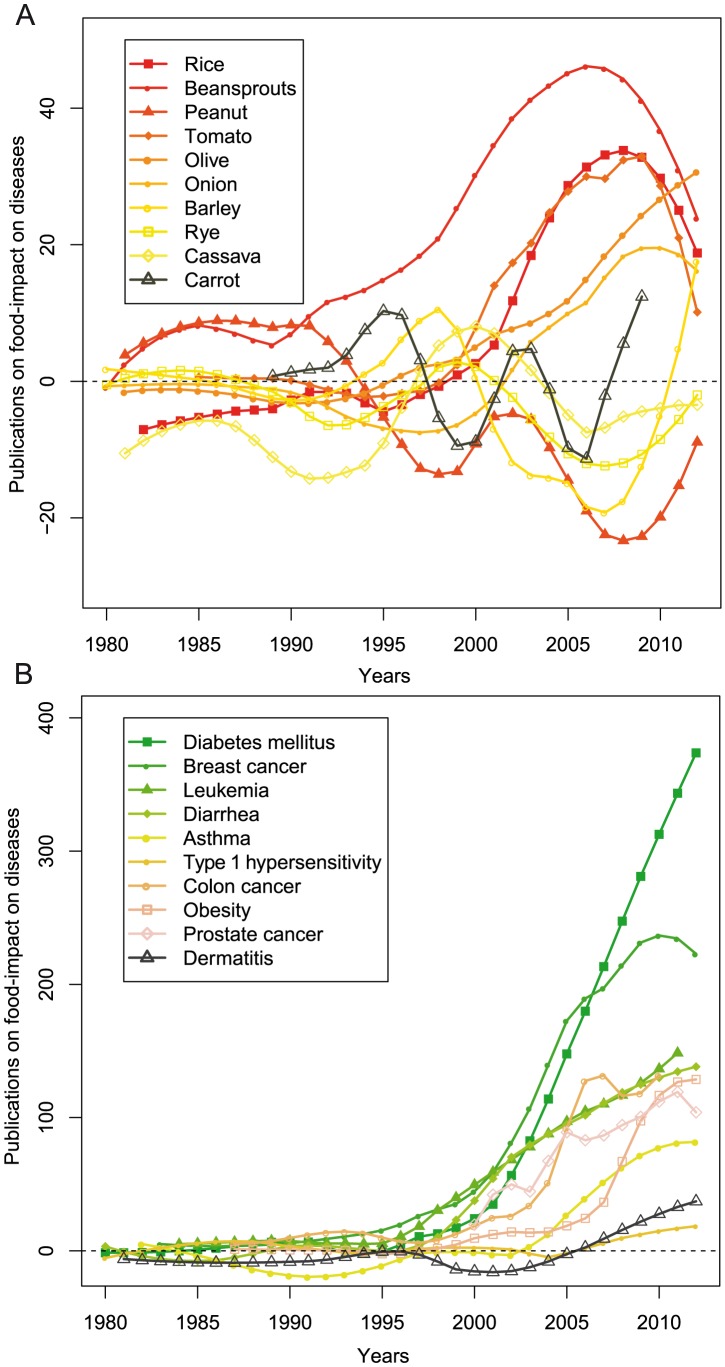
The association of foods with disease prevention/amelioration or disease progression throughout time. a) Examples of well studied foods in relation to positive (disease prevention/amelioration) and negative (disease progression) effect on health. The focus varies from negative effects (below 0) to positive effects (above 0) over the years. The value on the y-axis denotes the number of negative publications subtracted from the number of positive publications in a given year. b) Examples of well studied disease phenotypes in relation to food consumption. Likewise, the figure illustrates a change in focus from negative effects (below 0) to positives effects (above 0).

As shown in Figure 2A, research on the health effect of food effectively began in the early 80's and until middle 90's there was more research activity in relation to the negative effects of foods, such as their involvement in the development and progression of allergic reactions and asthma. However, the change of public opinion towards lifestyle and preventive strategies related to health in the last 15 years, resulted to an exponential growth of research papers reporting beneficial effects of plant-based foods against diabetes mellitus and different types of cancers (e.g. breast cancer, carcinoma and leukemia), not surprising since these diseases are the scourge of our time. Also of interest are the contradicting opinions over time on the health benefit of foods (Figure 2B). Until the beginning of the 21^st^ century there were only sparse reports on the health benefits associated with rice consumption, while the last 10 years there are numerous reports describing the positive impact of a rice-based diet. The opposite trend is observed for peanuts, which was mainly studied for its beneficial role in cancer before a number of studies begun correlating its consumption with health problems, such as allergy and hypersensitivity.

**Figure 3:**
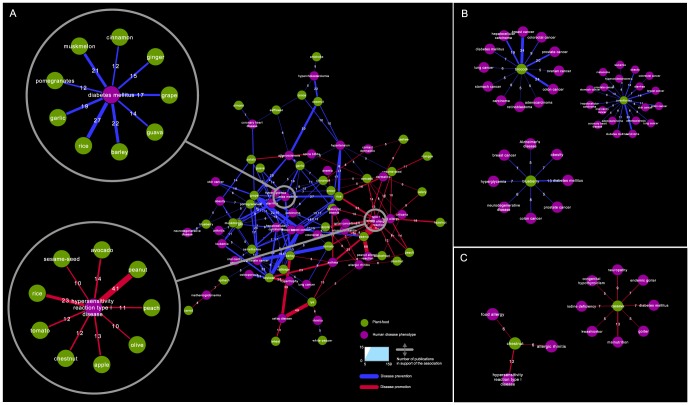
Food – disease association network. a) Disease phenotypes associated with common vegetables, fruits and plants of our diet. Foods are shown as green nodes and human disease phenotypes as purple nodes. Disease prevention/amelioration is depicted as a blue edge and disease promotion as a red edge. The size of the edge indicates the number of publications in support of the association. An edge is drawn between a food node and a disease node when there are at least five publications in support of this association. When a disease node has more than five edges, only the five strongest (with the most publication support) are shown on the network for the sake of clarity. Top left: zoom in the network formed between diabetes mellitus and foods that prevent/ameliorate the disease. Bottom left: zoom in the network formed between Type 1 hypersensitivity and foods that promote it. b) Examples of a vegetable (broccoli), a fruit (blueberry) and a plant-based beverage (camellia-tea) that are only positively associated with disease phenotypes. c) Two examples of foods that are only negatively associated with disease phenotypes.

The network of Figure 3A presents the most strongly supported associations of common foods and health benefits in the public literature. There are only a handful of common foods that have been associated either only positively or negatively with disease phenotypes. Consumption of broccoli, blueberry and camellia-tea for example, is consistently linked positively with a variety of disease phenotypes including diabetes mellitus, atherosclerosis and different types of cancers (Figure 3B). Cassava, a good source of carbohydrates but poor in protein, which constitutes the basic diet for many people in the developing world, has only negative associations with malnutrition, and malnutrition-related phenotypes (Figure 3C). For the majority of cases however, a particular food is positively correlated with specific disease phenotypes and negatively with others, highlighting the importance of personalized dietary interventions; rice is one characteristic example, associated positively to hypertension, diabetes, colon and breast cancer and negatively to dermatitis and hypersensitivity reactions. There are also several foods, including peanut, chestnut and avocado, consistently associated negatively with type-1 hypersensitivity and similar disease phenotypes, such as dermatitis, rhinitis and urticarial. Not surprisingly, a high number of publications exist for the negative effects of common foods such as wheat, barley and rye to celiac disease (also known as gluten intolerance). Figure 3 makes also evident that considerable research investments have been made in the past decades for enhancing our understanding of the association between diet and cancer; breast, prostate and colon cancers constitute the thickest edges on the network.

### Molecular level association of food to human disease phenotypes

**Figure 4:**
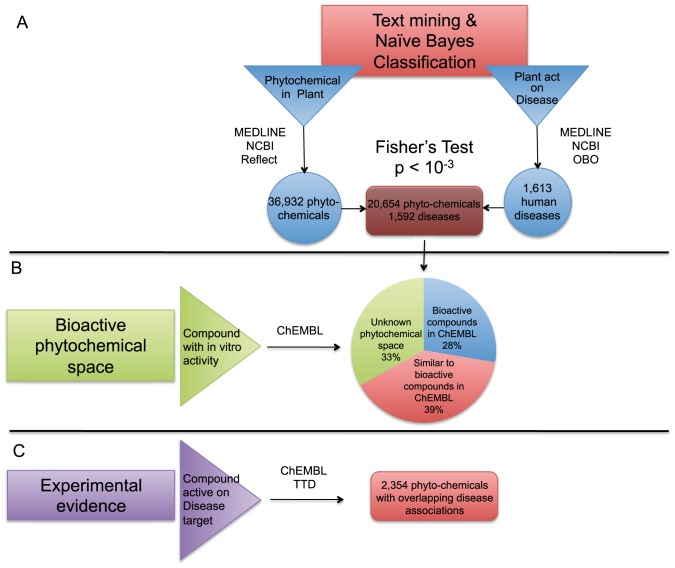
Association of phytochemicals to human disease phenotypes. The flow diagram illustrates the approach we followed for associating phytochemicals to human disease phenotypes. a) In the phytochemical - food and food - disease relations that we extracted by text mining, there are 7,077 plants with both phytochemical and human disease annotation. We used Fisher's exact test to identify statistically significant correlations between phytochemical and human disease phenotypes. At a 5% false discovery rate we identified 20,654 phytochemicals associated to 1,592 human disease phenotypes. b) 5,709 of the text-mined phytochemicals have been tested experimentally on a biological target and the activity data have been deposited in ChEMBL. For the remaining two thirds of the compounds, 8,113 phytochemicals are structurally similar to compounds with known protein targets (estimated with a Tanimoto coefficient >0.85), indicating similar bioactivity. The rest of the compounds, 6,832 phytochemicals, are not similar to any known bioactive compound and belong to a hitherto unexplored phytochemical space. c) We used the Therapeutic Targets Database to annotate the protein targets from ChEMBL to diseases. From the 5,709 phytochemicals that are included in ChEMBL, 2,354 are active against a biological target that is relevant for the same disease as the one we have predicted.

Our main hypothesis for the molecular level association of a plant-based diet to human disease phenotypes is that the positive or negative effect of a certain food on human health is due to the presence of one or more bioactive molecules in it. Towards this end, we used Fisher's exact test to systematically detect frequently occurring phytochemical - disease pairs through the phytochemical - food and food - disease relations that we extracted by text mining. At a 5% FDR we identified 20,654 phytochemicals connected to 1,592 human disease phenotypes, with approximately half of the disease associations being positive (Figure 4A).

Some of these phytochemicals have been previously studied *in vitro* for potential biological activity. By integrating information from ChEMBL we find that, from the 20,654 phytochemicals that the above analysis suggests as bioactive, approximately 5,709 have been tested experimentally on a biological target. From the remaining phytochemicals, for which no experimental bioactivity data are available, 8,113 compounds are structurally similar to compounds with known protein targets (estimated with a Tanimoto coefficient >0.85), indicating similar bioactivity, while the rest belong to a hitherto unexplored phytochemical space (Figure 4B).

In order to get an estimate of the performance of our approach to associate phytochemicals to diseases, we used the Therapeutic Targets Database to annotate the protein targets from ChEMBL to diseases. From the 5,709 phytochemicals that are included in ChEMBL, almost half are active against a biological target that is relevant for the same disease as the one we have predicted (Figure 4C).

Adding molecular-level information to food - disease associations allows us to zoom in the network of Figure 3 and generate lists of phytochemicals as promising drug-like candidates for subsequent target-based or cell line-based assay experiments, as we demonstrate in Table 1 with focus on a number of common cancer types. For example, 103 phytochemicals from 83 common foods [22]–[24] that through our analysis are associated with lung cancer, are structurally similar with 23 drugs from DrugBank that are approved for use in lung cancer treatment. In addition, by integrating information from ChEMBL and TTD, we identify 1,070 phytochemicals from 119 common foods with experimental activity against a lung cancer drug target. For cancer types, such as endometrial cancer and adenocarcinoma, where the drugs currently available in the market are scarce, this approach could be of particular interest, as it provides new opportunities for the identification of new drug candidates.

**Table 1:** Phytochemicals are associated with diseases via the approach illustrated in Figure 4.

Cancer type (DOID)	# drugs^1^	# associated phytochemicals similar to a drug	# associated phytochemicals with experimental disease-related target^2^	# common foods with disease-associated phytochemicals^3^
Breast cancer (1612)	44	344	1,840	94 (120)
Leukemia (162)	36	302	1,067	95 (118)
Lung cancer (1324)	23	103	1,070	83 (119)
Prostate cancer (10283)	20	170	2,105	82 (120)
Lymphoma (0060058)	20	146	527	80 (115)
Urinary system carcinoma (3996)	11	28	1,623	58 (121)
Ovarian cancer (2394)	11	15	1,219	49 (117)
Sarcoma (1115)	8	38	45	26 (82)
Intestinal cancer (10155)	8	52	1,530	86 (120)
Testicular cancer (2998)	7	41	0	51 (0)
Kidney cancer (263)	6	12	1,605	39 (121)
Melanoma (1909)	5	4	275	11 (114)
Renal cell carcinoma (4450)	5	8	1,271	30 (120)
Pancreatic cancer (1793)	4	28	1,331	53 (119)
Liver cancer (3571)	4	24	781	53 (118)
Skin carcinoma (3451)	2	8	11	16 (58)
Adenocarcinoma (299)	2	28	7	44 (19)
Endometrial cancer (1380)	2	97	20	58 (88)

For exemplary cancer types, we list the number of phytochemicals that are similar to small compound drugs that are approved for treatment of the disease (column 3), the number of phytochemicals that have experimental activity against a target implicated in this cancer type (column 4) and the corresponding number of common foods that contain these phytochemicals (column 5).

DOID: Human Disease Ontology Identifier.

1 from DRUGBANK.

2 from ChEMBL and TTD.

3 similar to a drug (with exp. disease-related target).

### Case study on colon cancer

To demonstrate the full potential of our approach we selected colon (colorectal) cancer as a case study and analyzed our results in the three directions shown below. Colon cancer is the second largest cause of cancer-related deaths in Western countries and various diet intervention and epidemiological studies suggest that diet is a vital tool for both prevention and treatment of the disease [3], [25].

**Figure 5:**
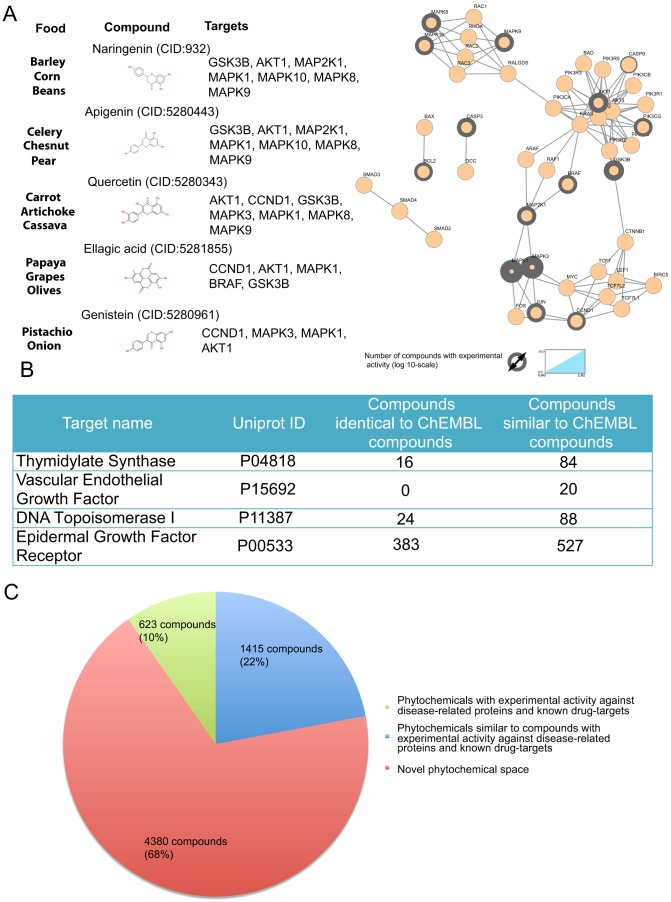
Targeting the colon cancer disease pathway with food components. a) The KEGG colon cancer disease pathway map is illustrated on the right, where the number of phytochemicals with experimentally measured bioactivity data is depicted as grey ring of varying width. Examples of bioactive phytochemicals are listed on the left, along with typical food source and biological target. b) Protein targets of typical colon cancer drugs and number of phytochemicals with experimental and predicted activities against them. c) From the 6,418 molecules associated with a health benefit for colon cancer, 623 have measured experimental activity against proteins from the colon cancer pathway or targets of colon cancer drugs. On the remaining phytochemical space linked to colon cancer, we can use chemoinformatics to predict activity based on compound structure and select the most promising candidates for *in vitro* or *in vivo* experimental validation. Accordingly, we have identified 1,415 phytochemicals with potential activity against colon cancer. For reasons of consistency with the disease pathway map, protein targets are given with their corresponding gene names.

1. **One stop legacy knowledge-shop**. When one embarks into studying the effect of food on colon cancer, it is useful first to get a systems view of the existing knowledge. This includes information about what types of foods and phytochemicals have already been tested in relation to colon cancer, which are their biological targets and how these activities affect the biological networks that consist the disease pathway. Such a systems view of the influence of dietary molecules associated to colon cancer is sketched in Figure 5A, based on the knowledge derived from our text mining approach that has been projected on the colon cancer pathway from the KEGG PATHWAY Database (http://www.genome.jp/kegg-bin/show_pathway?hsadd05210). By surveying our data resource we found 519 plants associated with a health benefit towards colon cancer. Statistical analysis of the data for frequently occurring phytochemical - disease pairs, reveals significant associations between 6,418 phytochemicals and colon cancer. Among the molecules associated with a health benefit for colon cancer, 623 of them have experimentally verified activity against proteins involved in the colon cancer pathway (nodes with a grey ring in Figure 5A). Naringenin, apigenin, quercetin, ellagic acid and genistein are examples of such compounds. Naringenin is commonly found in barley, beans and corn and apigenin is found in chestnuts, celery and pear. These foods have been associated with colon cancer prevention in a number of studies [26]–[28]. When tested *in vivo*, both compounds have been found able to suppress colon carcinogenesis [29]. In addition, in *in vitro* experiments naringenin and apigenin have seven targets on the KEGG colon cancer pathway. Quercetin, found in artichoke, carrot and cassava, and ellagic acid, present in grapes, papaya and olives have seven and five targets, respectively, on the KEGG colon cancer disease pathway, while genistein, found in pistachio-nuts and onions, has four. In most, if not all, of these cases, interest on the biological activity of the phytochemicals emerged after observations that the foods that contain them have some health benefit in relation to colon cancer prevention and treatment [30]–[32].
  Typical drugs in the market against colon cancer are listed in Figure 5B, along with their main protein targets. By surveying our data resource we identified a number of phytochemicals that have measured experimental activity against the same proteins. Riboflavin monophosphate, for example, which is found in many common foods such as almond, broccoli and tomato, is one among the 16 phytochemicals we have identified with biological activity against thymidylate synthase, the main target of drugs 5-fluorouracil and capecitabine [33]. Similarly, reserpine, a natural compound that has found applications as antihypertensive and antipsychotic, exhibits activity against DNA topoisomerase I [34] - the target of the colon cancer drug irinotecan - which could be interesting to investigate further in the light of drug repurposing.
2. **Discovery of novel bioactive compounds with drug-like properties.**As we saw above, from the 6,418 molecules associated with a health benefit for colon cancer, only 623 have experimentally verified activity against colon cancer protein targets (Figure 5C). On the remaining phytochemical space linked to colon cancer, we can use chemoinformatics approaches to predict activity based on compound structure and select the most promising candidates for *in vitro* testing. By encoding the structure in 2D fingerprints and setting a Tanimoto coefficient of 0.85 as the similarity threshold, 1,415 molecules turn up as structurally similar to a phytochemical or a synthetic compound from ChEMBL with activity against a protein from the colon cancer pathway or a colon cancer drug target (Figure 5B). The compounds listed in Table 2 are such examples, for which we can infer their bioactivity from experiments performed on structurally similar compounds.
  In regards to the remaining phytochemicals that our approach has associated to colon cancer, for which there exists no experimental protein target information and are not structurally similar with molecules that interact with colon cancer proteins, more advanced chemoinformatics techniques could be applied, such as pharmacophore-based similarity and docking. Alternatively, *in vivo* assays in model animals or *in vitro* experiments on disease cell lines could assist in elucidating their bioactivity. Such compounds with strong statistical support are beta-caryophyllene [35], guaiacol [36] and alloisoleucine [37] (p-value<10^−23^). Guaiacol, for example, has been identified in 93 plants in total, 32 of which are associated in the literature with colon cancer.
3. **Discovery of novel health benefits from foods.**One of the key observations from our analysis is that the majority of phytochemicals is found in a variety of foods, even in foods that are distant taxonomically. Thus, information about the bioactive phytochemical content of one food that has been characterized as beneficial towards colon cancer could help us identify other foods, which contain the same bioactive phytochemicals that may have similar health benefits. For example, cauliflower has been associated with a preventive effect on colon cancer [38], [39]. The adzuki bean shares 800 phytochemicals with it and could potentially have a similar effect on colon cancer as well; there exists, however, no such evidence in the literature. Such comparisons of phytochemical profiles could also find applications in the design of nutrigenomics studies, with the purpose to confirm that the study group follows a reference diet as different as possible from that of the control group, i.e. the two diets do not contain foods with similar phytochemical profiles.

**Table 2:** Phytochemicals (column 1) from common foods (column 2) with inferred activity to a colon cancer protein (column 3), based on structural similarity with an active compound from the ChEMBL library (column 4).

Compound name	# common foods	Predicted colon cancer target	Similar bioactive compound (Tc)	p-value for colon cancer
**Vanillin**	18	CCND1	CHEMBL53781 (0.86)	10^−23^
**Folic acid**	19	MAPK1, MAPK3, ERBB2	CHEMBL1679 (0.85)	10^−23^
**Spermidine**	16	CASP3	CHEMBL23194 (1.00)	10^−12^
**Vanillic acid**	27	MAPK1, MAPK3, ERBB2	CHEMBL32749 (0.88)	10^−12^
**Chalconaringenin**	1	JUN	CHEMBL129795 (0.86)	10^−11^
**Protocatechuic acid**	30	EGFR	CHEMBL145 (0.86)	10^−7^
**Quercetin-3-glucoside**	34	EGFR	CHEMBL486625 (0.85)	10^−4^
**Folinic acid**	1	TYMS	CHEMBL439741 (0.88)	10^−11^
**Protopanaxatriol**	4	TOP1	CHEMBL1096728 (0.85)	10^−3^

Listed compounds are examples of compounds predicted by our approach to have a positive effect against colon cancer, where p-values are included in column 5.

## Discussion

Food is a complex system that has an equally complex pattern of interactions with the human organism. As such, it consists the ideal platform for applying a systems biology approach, where different heterogeneous data sources are integrated and analyzed in a holistic way. Ferguson and Schlothauer in a review article that was published in 2012 [3] illustrated how information on the beneficial effect of broccoli against cancer is enriched by the integration of genomics, proteomics and metabolomics data. For a well studied food such as broccoli there is a rich body of evidence regarding its bioactive phytochemicals. Nevertheless, gathering and visualizing all evidence at once offered novel insights into the mechanisms by which broccoli may prevent cancer or retard cancer growth and progression.

An enormous scientific literature focusing on bioactive plant extracts and their phytochemicals, encompassing thousands of scientific papers, has emerged over the years. However, in order to utilize this wealth of information and integrate it with other types of data within systems biology studies, it is essential to first locate and then retrieve it in a high-throughput manner. The approach we have demonstrated here, which relies on the text mining of abstracts in PubMed/MEDLINE, has associated 23,137 phytochemicals with 15,722 plants, including approximately 2,768 edible fruits, vegetables and plant-based beverages. Even though there are several ongoing efforts that aim to collect information on molecular composition of food in a single resource, i.e. the Danish Food Composition Database (http://www.foodcomp.dk) centered on well-known organic nutrients, such as vitamins, amino acids, carbohydrates and fatty acids; the Phenol-Explorer [40] with information in text format for 500 polyphenols in over 400 foods and the KNApSAcK Family Database [17], these are rather limited in focus and size. For a molecular systems chemical biology approach of diet, the lack of chemical structures in the above databases is another significant bottleneck, as linking chemical names to a chemical structure in a high-throughput manner is not yet a straightforward process [41]. The most important contribution of our study is that it uses all the evidence generated during the last 100 years supporting health benefits of vegetables, fruits and other plants for establishing associations between foods, phytochemicals and human diseases, where entities from all three classes are annotated with unique, standard identifiers, so that they can be traceable in other databases. Moreover, chemical names and synonyms of all phytochemicals are linked to a unique chemical structure, which, besides traceability in other resources, allows for the application of chemoinformatics tools and their integration in systems chemical biology analyses. Last but not least, food associations to disease are annotated with directionality, which differentiates between causative and preventive effects of the food in relation to the specific disease.

Nevertheless, and despite the enormous amount of information collected here, we should also point out that inherent bias of meta-analysis allows for further improvements in our text mining pipeline. For example, while PubMed/MEDLINE is the most appropriate database for associating dietary interventions with disease phenotypes, it is certainly lacking scientific journals focused on the chemical composition of plants (for example, the Springer journal of Metabolomics; www.springer.com/lifesciences/biochemistry&biophysics/journal/11306). In order to overcome other common pitfalls of meta-analysis, such as data quality and data independence, it is our intention in the future to investigate the use of weighting parameters on the retrieved associations, so that, for example, associations generated from different labs constitute stronger evidence than associations from the same research team.

As we show in the case study on colon cancer, associating food, phytochemical content and diseases can build the basis for discovering novel bioactive compounds with drug-like properties. Furthermore, our analysis brought to the surface an undiscovered dietary component space of 8,113 phytochemicals that has not been previously linked to a health benefit and bears no structural similarities to other bioactive phytochemicals with established molecular targets. This represents a forthright opportunity for biochemists and nutritionists and offers a good basis for an attractive drug discovery platform.

At the same time, food safety authorities are concerned about the presence of compounds in herbal products and dietary supplements that could exert toxicity to humans [42]. For example, myristicin, a known component of nutmeg [43] and glycoalkaloids that are present in potatoes [44] can be extremely dangerous when taken in large doses. It is thus of great value to have *in silico* tools that are able to quickly list all phytochemicals associated to a given food in the public literature, and subsequently interrogate databases (e.g. the Comparative Toxicogenomics Database, http://ctdbase.org) for experimental evidence that associates the compounds in question or structurally similar compounds with a toxic effect.

Similar to research in the field of nutrition, scientists in ethnomedicine are seeking for evidence that can explain at the molecular level the health effect of traditional medicine. Ethnomedicine, such as Traditional Chinese Medicine and Ayurveda has existed and supported human health for thousands of years. A major barrier for developing an ethnomedicine evidence-based knowledgebase is that the current information related to plant substances for medicinal purposes is scattered and unstructured [45]. We provide a solution to this problem by extracting in a structured and standardized format phytochemicals that are associated with a medicinal plant, either in the open literature of the last 100 years or in the ethnomedicinal databases that we have *in-house*. Our approach facilitates the identification of novel bioactive compounds from natural sources and the repurposing of medicinal plants to other diseases than the ones traditionally used for, and builds a step towards elucidating their mechanism of action.

### Conclusion

Food is a factor that exerts influence on human health on a daily basis. Modulating the expression and the activity of enzymes, transcription factors, hormones and nuclear receptors is how food and its bioactive constituents modulate metabolic and signaling processes. The aim of our study is to provide the molecular basis of the effect of food on health in the complete spectrum of human diseases and to suggest why and how diet and dietary molecules may represent a valuable tool to reinforce the effect of therapies and protect from relapse.

Our systematized approach for connecting foods and their molecular components to diseases makes possible similar analyses as the one illustrated for colon cancer for approximately 2,300 disease phenotypes. In addition, it provides the phytochemical layer of information for nutritional systems biology studies with the aim to assess the systemic impact of food on health and make personalized nutritional recommendations.

## Methods

### Mining the literature for plant - phytochemical pairs

We retrieved the names of land plant species (embryophyta) and their synonyms from NCBI (http://www.ncbi.nlm.nih.gov/taxonomy). Chemical compound names and synonyms were taken from the argument browser Reflect [14]. With these two dictionaries the mining of 21 million titles and abstracts of PubMed/MEDLINE (http://www.nlm.nih.gov) was carried out using ChemTagger (https://pypi.python.org/pypi/ChemTagger). A Naive Bayes Classifier (https://pypi.python.org/pypi/NaiveBayes) was trained to recognize pairs of plants and phytochemicals.

A set of 200 tags, – plant and compound name entities – from 200 abstracts was compiled for training. As positive training set (PTS) we manually compiled a set of 75 abstracts mentioning plants and their phytochemical content. As negative training set (NTS) we manually compiled a set of 125 abstracts mentioning plants and chemical compounds, which we judged that did not refer to an actual plant - phytochemical content relationship. This includes, for example, abstracts that associate plants with synthetic small compounds in the context of chemical extraction and purification of plant extracts (e.g. ecdysonoic acid, 3-acetylecdysone 2-phosphate [46]) A feature vector was complied consisting of words within the abstract that were in proximity of each name-tag. The lexical features were chosen based on the term frequency–inverse document frequency (tf-idf) [47] and were sorted with the most frequent feature on the top and the least frequent at the bottom of the list. The training of the classifier commenced with only the highest score feature, while features with the next higher scores were added one by one, until the accuracy of the classifier stabilized at 31 features. Words such as “compound”, “isolated”, “extract” and “concentrated” were the features with the highest tf-idf score. Training was carried out using leave-one-out cross validation on the shuffled training data set. The performance of the classifier was subsequently evaluated on an external, balanced test set of 250 positive and negative abstracts, and resulted to 88.4% accuracy and 87.5% F1-measure. When the classifier was applied to the raw text of PubMed/MEDLINE, it retrieved 23,137 phytochemicals from 15,722 land-plant species (embryophyta) associated through 369,549 edges.

Chemical structures of the text-mined phytochemicals were retrieved from PubChem [48] ChEBI [49], CHEMLIST [50], the Chinese Natural Product Database [18] (CNPD) and the Ayurveda [19] that we have previously curated in-house [19]. Canonical SMILES were calculated with OpenBabel (http://openbabel.org/wiki/Canonical_SMILES) with no salts, isotopic or chiral center information. Edible plant names were retrieved from Plant For A Future (PFAF) (http://www.pfaf.org) and were mapped to NCBI IDs.

The taxonomy of plant species was retrieved from NCBI taxonomy (http://www.ncbi.nlm.nih.gov/taxonomy). Overrepresentation of phytochemicals on the taxonomy was calculated by using Fishers exact test, following the Benjamini-Hochberg procedure with a 5% false discovery rate [21]. A phytochemical that is significantly overrepresented on a specific class, order, family or genus of the taxonomy denotes that it is not randomly distributed over the whole tree. Since the association of plants to their phytochemicals was performed on the genus level, for this analysis we projected the phytochemical content of a child node to the parent node.

### Mining the literature for plant - disease associations

The names of land plant species (embryophyta) and their synonyms were taken from NCBI (http://www.ncbi.nlm.nih.gov/taxonomy). We retrieved 70,005 human disease terms and synonyms from the Open Biological and Biomedical Ontologies (OBO) Foundry [51]. The list of 143 common, non-processed foods was retrieved from the Danish Food Composition Database (http://www.foodcomp.dk). Names were mapped to NCBI land plant species and whenever *var.* IDs were available, they were subsequently collapsed to the corresponding species ID (e.g. broccoli and kale are varieties of the same *Brassica olerasea* species).

With these two dictionaries, text mining of 21 million titles and abstracts of PubMed/MEDLINE (http://www.nlm.nih.gov) was carried out using ChemTagger (https://pypi.python.org/pypi/ChemTagger).

A Naive Bayes Classifier (https://pypi.python.org/pypi/NaiveBayes) was trained to recognize pairs of plants and the associated human disease phenotypes. A set of 2,074 name-tags, plants and human disease phenotype name entities from 333 abstracts was compiled for training. Plants and human diseases with a ‘preventive’ association were used as the positive training set (PTS) and plants and human diseases with a ‘promoting’ association as the negative training set (NTS). Name entities of plants and human diseases mentioned in other contextual associations were used as the ‘noise’ training set (OTS).

For the training of the Naive Bayes Classifier, the lexical features were chosen based on the tf-idf score [47] and were sorted with the most frequent feature on the top and the least frequent at the bottom of the list. The training of the classifier commenced with only the highest score feature, while features with the next higher scores were added one by one, until the accuracy of the classifier stabilized at 71 features. Words such as “treatment”, “effect”, “patient”, “disease” and “plant” were the features with the highest tf-idf score. Training was carried out set using leave-one-out cross validation on the shuffled training data set. The performance of the classifier was subsequently evaluated on an external, balanced test set of 250 positive and negative abstracts, and resulted to 84.5% and an F1-measure of 84.4%. When the classifier was applied to the raw text of PubMed/MEDLINE, it retrieved 7,178 land-plant species associated with 1,613 human disease phenotypes through 38,090 edges. Plant - disease networks were constructed in Cytoscape v.2.8.1.

### Molecular level association of plant consumption to human disease phenotypes

We performed a categorical Fisher's exact test with the Benjamini-Hochberg procedure and a 5% false discovery rate [21] to associate particular phytochemicals with human disease phenotypes. Our alternative hypothesis was that the proportion of plants associated with a particular phytochemical is higher among the plants with a specific human disease phenotype than among those without. Our null hypothesis was that there is no relationship between plants associated with a particular phytochemical and a specific human disease phenotype.

Phytochemicals were associated to protein targets though experimental chemical-protein association data from ChEMBL, version 15 [52]. Canonical SMILES with no salts, isotopic or chiral center information (http://openbabel.org/wiki/Canonical_SMILES) were used as the unique molecular identifier for searching for common small compound entities between the phytochemical and ChEMBL lists. Human proteins were associated to diseases through the Therapeutic Targets Database [53] (TTD Version 4.3.02). Disease names were mapped to the OBO Foundry human disease ontology and ordered in disease categories. Disease pathway networks were constructed in Cytoscape v.2.8.1.

### Case study on colon cancer

The colon cancer disease pathway was obtained from KEGG PATHWAY Database (http://www.genome.jp/kegg-bin/show_pathway?hsadd05210). The network was constructed in Cytoscape v.2.8.1. Phytochemicals were associated to the proteins from the disease pathway though experimental chemical-protein association data from ChEMBL, version 15 [52]. Canonical SMILES with no salts, isotopic or chiral center information (http://openbabel.org/wiki/Canonical_SMILES) were used as the unique molecular identifier for searching for common small compound entities between the phytochemical and ChEMBL lists. Colon cancer drugs were obtained from KEGG Disease Entry: H00020 (http://www.genome.jp/dbget-bin/www_bget?ds:H00020) and their respective protein targets from the Therapeutic Targets [53] (TTD Version 4.3.02).

## Supporting Information

Figure S1 **Mapping the phytochemical space on the plant taxonomy.** 37,351 phytochemicals were mapped on the plant taxonomy. Only 8% of the recorded phytochemicals show localized enrichment (*p*-value<10^−4^). The taxonomy of land-plant species (embryophyta) was retrieved from NCBI taxonomy (http://www.ncbi.nlm.nih.gov/taxonomy). Nodes represent Classes (yellow), Orders (blue), Families (green) and Genera (pink) of the taxonomy tree. Links are placed between a parent and a child node, if they share conserved phytochemicals. A phytochemical is conserved, when it is overrepresented on both the parent and the child nodes. The width of the link corresponds to the number of conserved phytochemicals between parent and child nodes. The size of the node corresponds to the number of overrepresented phytochemicals on a given class, order, family or genus.
(EPS)

Table S1 List of phytochemicals described as SMILES that are localized on a taxonomy class, order, family or genus.
(XLS)
