# Correction: Integrated Text Mining and Chemoinformatics Analysis Associates Diet to Health Benefit at Molecular Level

**DOI:** 10.1371/annotation/96a702bd-85a5-49d9-8fcc-3aad7aa4afa7

**Published:** 2014-01-23

**Authors:** Kasper Jensen, Gianni Panagiotou, Irene Kouskoumvekaki

The affiliation for the third author is incorrect. Irene Kouskoumvekaki is not affiliated with #2 but with #1 Center for Biological Sequence Analysis, Department of Systems Biology, Technical University of Denmark, Kemitorvet, Lyngby, Denmark

